# Multiple fields manipulation on nitride material structures in ultraviolet light-emitting diodes

**DOI:** 10.1038/s41377-021-00563-0

**Published:** 2021-06-16

**Authors:** Jinchai Li, Na Gao, Duanjun Cai, Wei Lin, Kai Huang, Shuping Li, Junyong Kang

**Affiliations:** Engineering Research Center of Micro-nano Optoelectronic Materials and Devices, Ministry of Education, Fujian Key Laboratory of Semiconductor Materials and Applications, CI center for OSED, College of Physical Science and Technology, Xiamen University, 361005 Xiamen, China

**Keywords:** Inorganic LEDs, Metamaterials

## Abstract

As demonstrated during the COVID-19 pandemic, advanced deep ultraviolet (DUV) light sources (200–280 nm), such as AlGaN-based light-emitting diodes (LEDs) show excellence in preventing virus transmission, which further reveals their wide applications from biological, environmental, industrial to medical. However, the relatively low external quantum efficiencies (mostly lower than 10%) strongly restrict their wider or even potential applications, which have been known related to the intrinsic properties of high Al-content AlGaN semiconductor materials and especially their quantum structures. Here, we review recent progress in the development of novel concepts and techniques in AlGaN-based LEDs and summarize the multiple physical fields as a toolkit for effectively controlling and tailoring the crucial properties of nitride quantum structures. In addition, we describe the key challenges for further increasing the efficiency of DUV LEDs and provide an outlook for future developments.

## Abstract (web summary)

This paper review recent advances in ultraviolet LEDs and summarize that multiple physical fields could built a toolkit for effectively controlling and tailoring crucial properties of nitride quantum structures.

## Graphical abstract

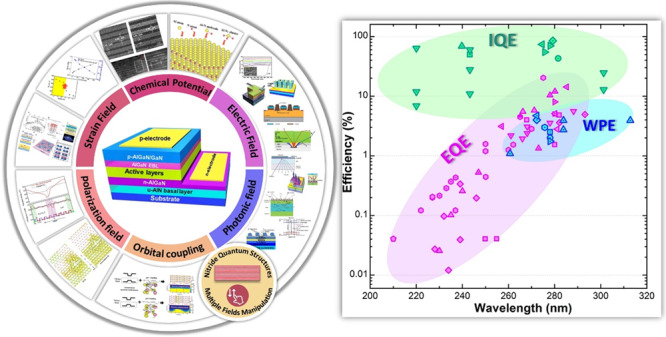

## Introduction

Recently, the COVID-19 pandemic has caused the outbreak of a global public health emergency. Until November 2020, more than 57 million cases, with more than 1.3 million deaths, have been confirmed. Furthermore, this ongoing disaster has led to a social and economic disruption globally, which widely raises awareness about public health and stimulated further discussion on the control means of disease transmission^1–4^. As we know, COVID-19 spreads from person to person mainly via the respiratory route with the exhalation of virus-containing particles, respiratory droplets, or aerosols, from an infected person^5^. Indirect contact via a contaminated surface or object could also largely enhance the spread of the virus^6–9^. Strategies for preventing infection include inoculating vaccines and blocking the route of disease transmission. Until the widespread availability of highly effective vaccines, preventing virus transmission is crucial. The recommended preventive measures include social distancing, wearing masks, washing hands, and disinfecting fomites^10–12^. Surfaces can be decontaminated by chemical solutions, such as 70% ethanol, 0.1% sodium hypochlorite, or 0.5% hydrogen peroxide^13^, or by germicidal irradiation with deep ultraviolet (DUV) light (200–280 nm)^14,15^.

**Fig. 1:**
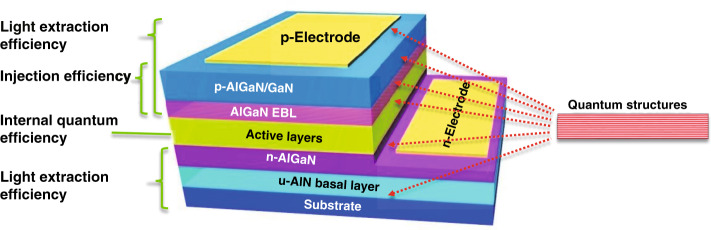
Schematic of an AlGaN DUV LED structure. Relative layers are contributed to the main efficiency parameters including the injection, internal quantum, and extraction efficiencies. The AlGaN quantum structures have been implanted into heterostructural epilayers for the efficiency improvements

DUV radiation with high energy is known to be able to damage a microorganism’s DNA or RNA, including bacteria, spores, and viruses, by changing its nucleic acids, thereby its ability to reproduce can be partially or fully impaired^16–18^. The germicidal effectiveness curve peak is ~265 nm^19^. However, the natural solar ultraviolet light is largely blocked by the atmosphere (by 77%), and only a small fraction of DUV reaches the ground. Hence, the available DUV light derives mainly from artificial sources, such as mercury lamps, excimer lamps, and light-emitting diodes (LEDs). Mercury and excimer lamps, which are traditional sources, are large, toxic, unstable, and short lifetimes; in contrast, DUV LED has proved its remarkable advantages as well as potential applications in many fields, especially in disinfection and sterilization^20–25^. Recent researches have revealed that DUV light at 207–222 nm has significant potential to kill pathogens without damaging exposed human tissues and can be a sterilization light source that is harmless to human skin and eyes^26,27^. After decade’s efforts, the level of the external quantum efficiency (EQE) of most commercial and laboratorial DUV devices still remains below 10%^28,29^ (see also Fig. 14). Furthermore, the EQE dramatically decreases to approximately 1% and 0.1% when the emission wavelength is below 260 and 230 nm, respectively^30,31^. Such a low efficiency strongly restricts the range of the potential applications of DUV LEDs. Originally, the challenges to improve their performances could be attributed to systematic and interrelated difficulties in the whole structure of LED devices from the substrate, AlN basal layer, *n*- and *p*-type layers, active layers, up to contacting electrodes (Fig. 1)^32^. The further increase of the injection, radiative, extraction, and electrical efficiencies (Fig. 1) becomes necessary to enhance the performances with high EQE of AlGaN-based DUV LEDs.

Owing to the fast scale-down of the structural size of advanced materials and the rapid development of epitaxial instruments and techniques, quantum structures gradually exhibit their unique advantages over the traditional device structure of semiconductors and have been widely implanted into DUV LEDs (Fig. 1). The internal quantum efficiency (IQE) is mainly related to the quality of the active layers with quantum structure, such as single quantum wells (SQWs) and multi-quantum wells (MQWs). In principles, the scale of semiconductor quantum structure is only a few nanometers. Its growth can usually be accomplished under non-equilibrium conditions, where the growth kinetics appears very complicated and dependent on the field of chemical potentials of molecules. The pre-reaction of precursors, the adsorption, diffusion, and desorption on the substrate are subjected to extremely complicated parameters. As we know, the cohesion of Al atoms and the difficulty of their migration on the substrate surface strongly restrict the improvement of the quality of MQWs^33^. On the other hand, because the III-nitrides possess large spontaneous and piezoelectric polarization, the polarization electric fields in MQWs separate carriers for effective radiative recombination^34–36^. Meanwhile, the heteroepitaxy and heterostructure inevitably subject AlGaN layers to large and complicated strain fields^37^, this strongly affects the crystal quality and causes the piezoelectric fields. Therefore, the carrier confinement in quantum structures plays a key role in overlapping carriers against polarization field and in the operation of optoelectronic devices^38,39^. When the quantum structure is reduced to the atomic scale, lattice discontinuities must be taken into account. The carrier injection efficiency is closely related to the quality of the conductive layer, e.g., the net carrier concentration in the *n*- and *p*-type conductive layers. For III-nitrides, *p*-type doping is much more difficult than *n*-type doping. In the case of GaN, the activation energy of *p*-type-doped Mg acceptor is as high as ~160 meV, thereby resulting in a hole concentration lower than the electron concentration by 1–2 orders of magnitude^40^. This problem becomes much serious as the Al content in AlGaN increases. It has been proved that a low Mg doping concentration in AlGaN materials is highly relative to the higher formation energy of Mg impurities^40^ and the activation energy of Mg acceptor increases linearly (465–758 meV in AlN)^41^. The light extraction efficiency (LEE) is closely related to the refractive index of the material and the optical fields. Generally, photons emitted from the active layer of a DUV LED must propagate out of the device to form effective lighting. However, light will be reflected at the interface between media and will be absorbed by *p*- or *n*-type layers and electrodes. For AlGaN, the total reflection angle is only 26°^42^, thereby resulting in extremely low LEE. On the other hand, AlGaN materials with high Al content have significant optical anisotropy. The emitted light from the active layer has a much larger transverse magnetic (TM) polarized portion^43,44^, which propagates laterally towards the sidewall of *c*-plane AlGaN epilayers. This means that most of the light emission cannot be extracted out of the top face of the device.

**Fig. 2:**
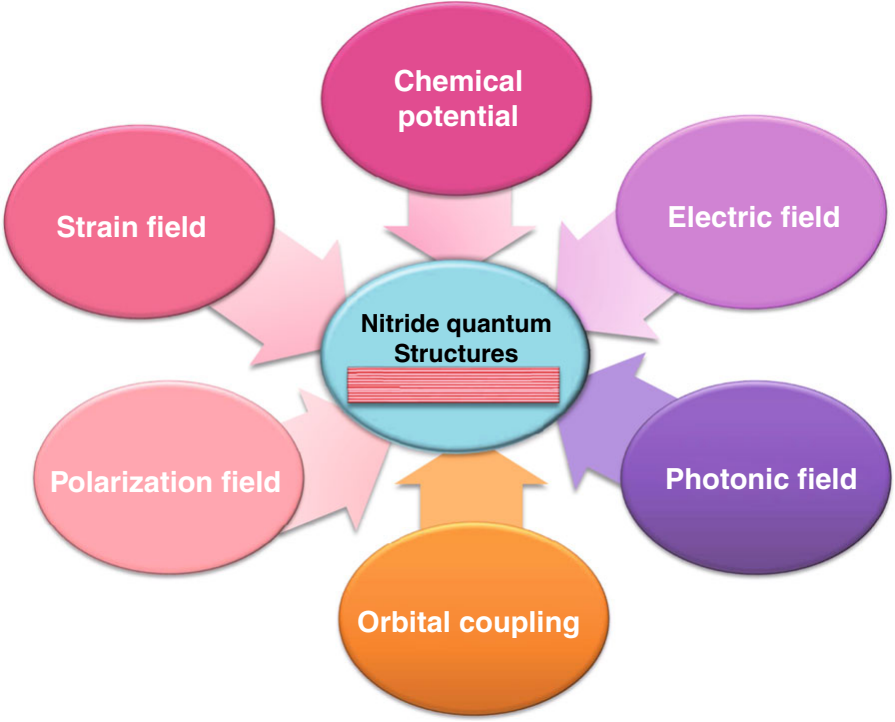
Schematic of the impact of multiple physical fields on nitride quantum structures. These tunable multiple fields include strain field, electric field, optical field, electromagnetic field, the field of chemical potential, orbital coupling, etc.

From the aforementioned facts, one can realize that in various parts of the DUV device, within critical quantum structures, and on crucial problems, multiple physical fields have been proved important in affecting, controlling, and even adjusting the properties of nitride quantum structures, the performance of devices, and the behaviors of various particles, as illustrated in Fig. 2. Hence, to overcome the efficiency bottleneck of the AlGaN-based DUV light sources includes not only simple technical issues but also deep scientific problems. After decades of efforts by worldwide researchers in this community, the features of these physical fields have been revealed and could be summarized into a toolkit for intentional tuning of the properties of nitride quantum structures. Once the expected performance of AlGaN-based DUV LEDs is achieved, the relative application market could explode rapidly.

## Manipulation of fields of chemical potentials

**Fig. 3:**
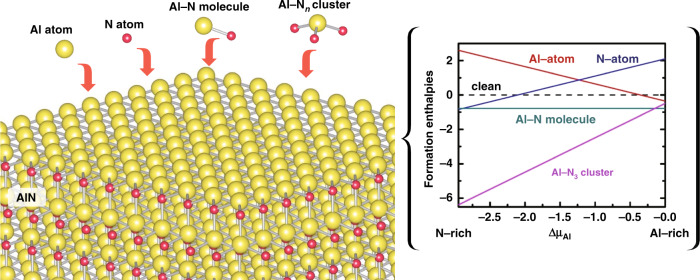
Formation enthalpies of the AlN surfaces with an Al atom, N atom, Al–N molecule, and Al–N_3_ cluster adsorption as the function of the Al chemical potential. Figures reproduced from ref. ^57^, © 2013 American Chemical Society

One of the most fundamental and crucial issues is to improve the crystal quality of AlN basal layers. Starting with the substrate, systematic works have addressed the buffer techniques beneath the AlN epilayer, including reactive plasma deposited AlN nucleation layers^45,46^, low/high-temperature AlN buffer layers^47^, double AlN buffer layers^48^, superlattice (SL) buffer layers^49^, microtrenches^50,51^, nanopatterned sapphire substrates^52^, and nanopatterned AlN buffer layers^48^. In the growth process, epitaxial strategies have been proposed as migration-enhanced metal-organic chemical vapor deposition (MOCVD)^53^, migration-enhanced lateral epitaxial overgrowth of AlN^50^, and multilayered AlN^54–56^. However, the realization of AlGaN with atomically abrupt surfaces and/or interfaces is still challenging in MOCVD technique. From the viewpoint of the microscopic growth mechanisms with basic constituent units, including the Al/N atoms, Al–N molecule, and Al–N_3_ cluster, the different migration behaviors strongly depend on the field of their chemical potentials (Fig. 3), which allows for using hierarchical growth units via appropriate control and choice of precursors in the growth process. In this process, the AlN epilayers could be grown with more compact and smoother surface morphologies as well as optimized crystal qualities^57^. To shift the DUV emission towards shorter wavelength with efficient light extraction from the top face of the device, the construction of GaN/AlN quantum structures has become a widely concerned issue for the replacement of high-Al-content AlGaN alloys. Aiming at the precise tailoring of critical parameters of the AlN and GaN heterostructures, the digitally stacked GaN/AlN structure, i.e., short-period GaN/AlN SLs, has been proposed. The short period indicates the extremely abrupt and ultrathin well and barrier layers with a thickness of just a few atomic layers. For such an advanced structure, the coherent lattice, abrupt interface, and rapid alternation are of great significance in the growth technique.

Researches on the growth of GaN/AlN short-period SLs was pioneered by Khan et al. in 1990s. The switched atomic layer epitaxy yields a sharp absorption edge and clear interfaces^58^. Following works on this issue further revealed that high-quality GaN/AlN short-period SLs possess properties that are significantly different from traditional continuous AlGaN epilayer^59–62^. In 2011, Rodaka et al. worked on AlN/GaN SLs and explored the relationship of the binary alloy growth rates with the interfacial quality^63^. Taniyasua et al. employed the GaN/AlN short-period SLs on SiC substrates as the active layer for DUV LEDs. By decreasing the GaN well thickness from 2.5 monolayers (MLs) down to 0.9 ML via the MOCVD method, they achieved a short-wavelength emission at 236.9 nm from the *c*-plane surface^64^. In 2014, the GaN/AlN short-period SLs with sharp interfaces grown by plasma-assisted molecular beam epitaxy (PA-MBE) was demonstrated by Kuchuk et al., however, these SLs showed compositional fluctuation and non-uniform random distribution^65^.

Although MBE method provides high controllability of short-period SLs with atomic layer-by-layer epitaxy, the slow growth rate makes it unsuitable for industrial productions. The medium growth rate enables MOCVD an ideal method for typical nitrides growth. However, the epitaxial growth in MOCVD actually is under non-equilibrium conditions, which include extremely complicated kinetics and dynamic processes. Especially for the AlN/GaN heterostructural epitaxy, challenges lie in the control of the decomposition and pre-reaction of MO precursors as well as the kinetic processes of deposition, such as adsorption, diffusion, and desorption on the substrate surface^66–68^. On the other hand, the Al atom has a high adhesion coefficient and slow migration velocity due to the limit of meddling reaction temperature^69^. How to overcome these problems attracted broad interests of researchers in this community.

**Fig. 4:**
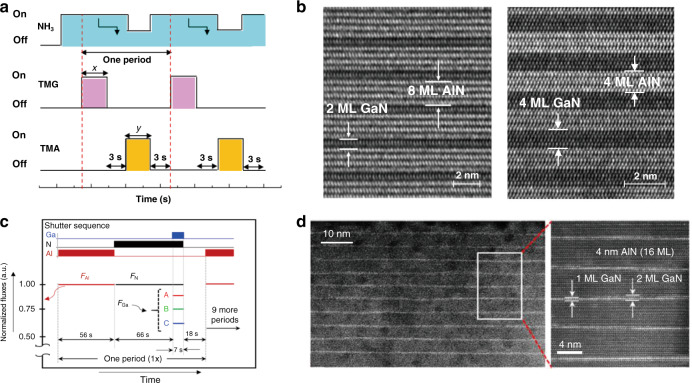
Schematic sequence and the cross-sectional morphologies for the grown short-period (AlN)_m_/(GaN)_n_ SLs. **a** MOCVD schematic sequence and **b** high-resolution cross-sectional TEM images for the hierarchical growth units of (AlN)_*m*_/(GaN)_*n*_ short-period SLs with different well and barrier thicknesses. **c** MBE growth diagram showing shutter sequence for a single period of the growth and **d** the Z-contrast STEM images for the 10 periods of GaN/AlN heterostructures. Figures reproduced from **a** and **b** ref. ^71^, © 2019 American Chemical Society; **c** and **d** ref. ^73^, © 2017 AIP Publishing

Based on the hierarchical growth of AlN epilayers, the atomically tunable well and barrier layers in the short-period (AlN)_*m*_/(GaN)_*n*_ SLs were grown on AlN/sapphire template by Gao et al. in 2014^70^. By switching the growth sequence instantaneously, the short-period AlN/GaN SLs wells achieve coherent growth. Clear and atomically abrupt interfaces, as well as single atomic layers of GaN, were recognized. In 2019, Gao et al. further demonstrated the underlying growth mechanism of constituent elements during the formation of the digital alloyed integral MLs revealed that the extreme circumstance of the nitrogen-rich condition could effectively stabilize the nitrogen adatoms with higher smoothness on the Ga-terminated interface. Al-rich condition favors the formation of Al layer while the deposition of Ga adatomic layer appears insensitive to the atmosphere. Based on these principles, the manipulation of the fields of chemical potentials was proposed to grow constituent elements of AlN and GaN layer-by-layer (Fig. 4a). The precisely integral MLs with atomic flatness and abrupt interfaces have been achieved without observable compositional fluctuations, as shown in Fig. 4b. The concept of chemical potential manipulation strongly indicates a practical scheme for the precise controlling of the extreme quantum structures under non-equilibrium growth conditions, e.g., in the MOCVD system.

In 2016, Rong et al. proposed a novel GaN/Al_0.75_Ga_0.25_N structure with quasi-two-dimensional (2D) GaN layers inserted into AlGaN matrix by MBE, which acted as the active region for high-efficiency electron-beam pumped mid-UV (285 nm approximately) light sources^72^. By further optimizing MBE growth conditions to achieve atomic layer precision, GaN/AlN quantum heterostructures were successively epitaxially grown by Islam et al. (Fig. 4c–d), who demonstrated the 230–270 nm electroluminescence (EL) emission from DUV LEDs in 2017^73,74^. In 2019, Shan et al. reported the DUV laser emitting at 249 nm by optical pumping based on binary AlN/GaN heterojunctions, which was comparable to state-of-the-art AlGaN quantum well (QW) lasers at similar wavelengths^75^. In 2020, Toropov et al. demonstrated an enhancement of the short-range electron–hole spin-exchange in GaN/AlN structure with the embedded single GaN well and reported the 2D exciton nature of light emission at temperatures up to 300 K with a possibly short emission wavelength^76^.

## Manipulation of strain fields

On account of the heteroepitaxial growth and heterostructural construction, AlGaN epilayers and quantum structures are inevitably subjected to large misfit strains^77^. This has been well known as a fundamental situation of AlGaN materials and related devices. Recently, research works have been conducted to minimize the influence of misfit strain by releasing it through various techniques. Furthermore, the stress field within the AlGaN quantum systems has gradually been considered and utilized as an operable tool to manipulate the structural and optoelectronic properties of their functional structures and advanced devices.

For misfit strain release, there have been three important research branches: the gradient stress field methods through epitaxial lateral overgrowth (ELOG) techniques, the multi-period SLs inserting layers, and the van-der-Waals epitaxial growth with buffering by 2D materials.

**Fig. 5:**
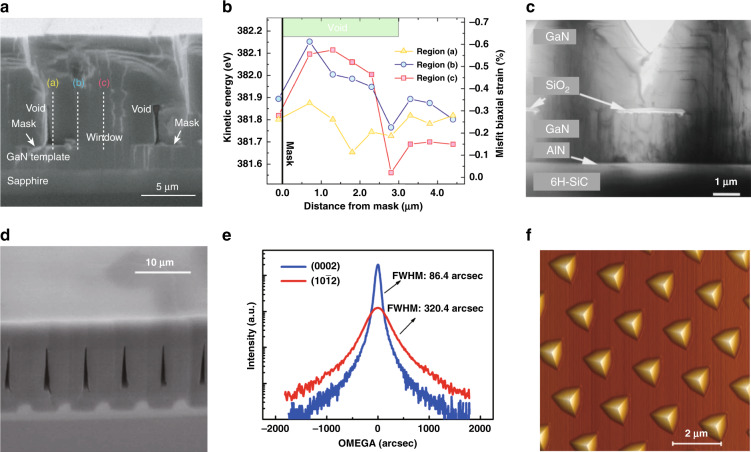
Strain field relaxation through ELOG method. **a** SEM image of the cross-section of ELO GaN. **b** Misfit biaxial strain distributions in ELO GaN by AES. **c** Cross-sectional TEM micrograph in [11$\overline 2$0] orientation from a selectively grown GaN stripe revealing the coalescence and change of the direction of dislocation lines. **d** Cross-sectional SEM micrograph of a fully coalesced 20â€‰μm-thick AlN film grown by migration enhanced lateral epitaxial overgrowth technique. e XRCs of (0002) and (10$\overline 1$2) diffractions for the AlN films grown on the nanopatterned substrate. **f** 3D AFM image for the surface morphology of the pyramidal patterned sapphire substrate. Figures reproduced from **a** and **b** ref. ^81^, © 2005 AIP Publishing; **c** ref. ^78^, © 1997 AIP Publishing; **d** and **e** ref. ^84^, © 2013 AIP Publishing; **f** ref. ^85^, © 2019 AIP Publishing

The ELOG concept and related technique were first proposed in 1997 and applied in GaN epitaxial growth, thereby effectively proving the crystal quality by lowering the density of threading dislocations (TDs) (Fig. 5c)^78,79^. In ELOG method, the crucial technique is to pattern the template or substrate with dielectric mask or etched trenches, which could allow the selected area overgrowth of epilayer above. Afterward, a lateral overgrowth could be achieved by enhancing the growth laterally and coalescing over the mask or void^80^. In 2005, Cai et al. established a novel scheme based on Auger electron spectroscopy (AES) system for high-spatial-resolution strain measurement (in nanometer scale) and investigated the strain field distribution on ELOG area (Fig. 5a, b)^81^. It has been found that, together with the bending of TDs, a crucial stage for strain release could occur within a distance range above the mask, thereby leading to the turning of the propagation direction of TDs laterally^82^. This is regarded as the main reason for the release of misfit strain and the improvement of GaN epilayer crystal quality. Thereafter, the ELOG technique has been applied to the AlN epitaxy and extended to nano-patterned substrates. In 2007, Asif Khan et al. showed that micro-stripe-patterned sapphires or AlN/FSS templates could effectively enhance the light output power of DUV LEDs by reducing the TDs^83^. In 2008, Jain et al. reported on the growth of low-defect thick films of AlN and AlGaN on trenched AlGaN/sapphire templates using migration enhanced lateral epitaxial overgrowth (and modified pulse growth) (Fig. 5d)^50^. To decrease the coalescence thickness, in 2013, Yan et al. employed a nanosphere lithography method to fabricate nano-patterned sapphire substrates for the ELOG of AlN epilayer and achieved an AlN coalescence thickness of only 3 μm (Fig. 5e)^84^. Meanwhile, it also leads to the low dislocation densities in AlN and epilayers above. In 2019, Chen et al. demonstrated a crack and strain-free AlN epilayer with a thickness of 10.6 μm grown on a pyramidal-patterned sapphire substrate (Fig. 5f)^85^. The full width at half-maximum (FWHM) of the X-ray rocking curve was 165/185 arcsec for (002)/(102) planes, respectively. A dual coalescence of the AlN epilayer was observed, which can effectively relax strain during the heteroepitaxy process. In 2020, Hagedorn et al. reported an 800 nm-thick, fully coalesced, and crack-free AlN grown on two-inch hole-type nanopatterned sapphire wafers by high-temperature annealing (1680 °C) method^86^.

**Fig. 6:**
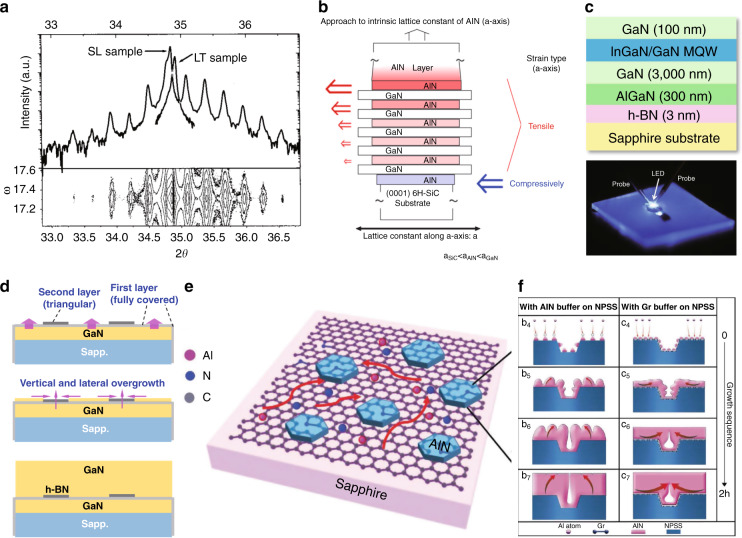
Van der Waals epitaxy for misfit strain release. **a** XRD scans of the 3.0 mm SL and low-temperature buffer AlN samples (upper) and (0002) mapping of the SL sample. **b** Model of strain control of the AlN template by the introduction of the (AlN/GaN) multi-buffer layer. **c** (Upper) Single-crystal h-BN release layer growth on sapphire for AlGaN MQWs and (lower) optical image of the blue light EL from the transferred vertical-type LED. **d** Schematic diagram illustrating the overgrowth process of GaN thick epilayer on the h-BN buffered GaN/sapphire template. **e** Schematic diagram of the nucleation and film growth of AlN on N_2_-plasma-treated graphene/sapphire substrate. **f** Schematic illustration of morphology evolution for AlN films grown with low-temperature AlN buffer and graphene buffer, respectively. Figures reproduced from **a** ref. ^87^, © 2002 AIP Publishing; **b** ref. ^88^, © 2007 Elsevier; **c** ref. ^92^, © 2012 Springer Nature; **d** ref. ^93^, © 2016 Springer Nature; **e** ref. ^94^, © 2014, American Chemical Society; **f** ref. ^96^, © 2020, John Wiley and Sons

SL is another quantum structure with very short-period QWs. In 2002, Asif Khan et al. revealed that the insertion of a set of AlN/AlGaN SLs (Fig. 6a) could significantly reduce the biaxial tensile strain, thereby resulting in 3-mm-thick, crack-free Al_0.2_Ga_0.8_N layers^87^. It was also observed that the TDs could merge in the SLs region and consequently, the density of TDs is reduced greatly. In 2007, Niikura et al. achieved AlN and AlGaN epitaxial layers with Al composition ranging from 0.6 to 0.8 on a (0001) 6H–SiC substrate using the (AlN/GaN) multi-buffer layer structure (Fig. 6b)^88^. The strain of the grown layer could be controlled by the structure of the inserted (AlN/GaN). It was found that the crystal quality of the grown layer could be improved by increasing the tensile strain in *a*-axis (compressive strain in *c*-axis). The FWHM of (0002) and (10$\overline 1$2) were decreased to 79 and 853 arcsec, respectively.

In recent years, 2D materials, such as graphene and hexagonal boron nitride (h-BN) are emerging advanced materials with various breakthroughs, which exhibit unique properties and functionalities^89–91^. Owing to their week out-of-plane van der Waals interaction, several pioneering works have been conducted on misfit strain release and quality improvement of AlN and AlGaN epilayers and quantum structures. In 2012, Kobayashi et al. first demonstrated that the h-BN can form a release layer that enables the mechanical transfer of GaN-based device structures onto foreign substrates (Fig. 6c)^92^. In 2016, Cai et al. achieved a large-roll synthesis of monolayer h-BN film by CVD method and presented the overgrowth of thick GaN wafer over 200 μm through the van der Waals epitaxy with h-BN buffering, free of residual strain (Fig. 6d)^93^. In 2018, Qi et al. utilized graphene as a buffer layer for the growth of an AlN film on a sapphire substrate and revealed the relaxation of compressive strain as well as the reduction TDs in AlN epilayer (Fig. 6e)^94,95^. In 2020, Wei et al. further showed that the AlN grown on graphene will prefer the lateral growth and quick coalescence on the nano-patterned substrate, resulting in low strain and low dislocation density (Fig. 6f)^96^.

**Fig. 7:**
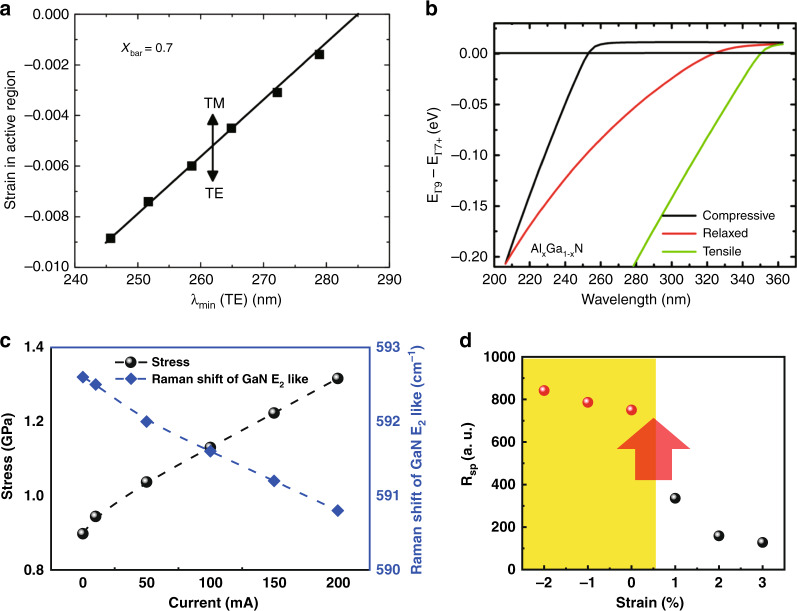
The strain field effect on the energy band structure, transition and emission in AlGaN quantum structures. **a** The strain in active region required to achieve TE polarization at prescribed wavelengths. **b** Calculated energy spacing between the Г_9_ and Г_7+_ VBs for fully compressive AlGaN grown on AlN, fully relaxed AlGaN, and fully tensile AlGaN grown on GaN. **c** The biaxial stress of AlGaN MQWs derived from stress-free Al_0.52_Ga_0.48_N as a function of current. **d** The calculated total spontaneous emission rate as a function of strain. Figures reproduced from **a** ref. ^97^, © 2012 AIP Publishing; **b** ref. ^98^, © 2015 AIP Publishing; **c** and **d** ref. ^103^, © 2017 The Royal Society of Chemistry

Based on these achievements, it has been realized that the strain field could be intentionally managed for AlGaN quantum structures, aiming at energy band engineering, transition controlling, and emission tuning. In 2012, J. E. Northrup et al. reported that the polarization of the light emitted from DUV-LEDs can be controlled by engineering the strain state in the active region (Fig. 7a)^97^. The compressive strains lead to a reordering of the valence sub-band of AlGaN quantum structures and consequently the enhancement of the degree of light polarization (Fig. 7b)^98^. To modulate the strain of the AlGaN quantum structures, numerous attempts have been proposed. Highly compressively strained QWs were realized by using AlN bulk or patterned AlN/sapphire as substrates owing to the differences of thermal expansion coefficients and the coalescence process^99,100^. In 2018, Long et al. proposed a method for increasing the compressive strain of MQWs by inserting an underlying *n*-AlGaN layer, thereby enhancing the PL intensity^101^. In 2020, Zhang et al. adopted multiple alternation cycles of low- and high-temperature growth to modulate the strain state of the AlN template, and the polarization degree of AlGaN QWs effectively increased from 41.5% to 61.9%^102^. In addition to these growth conditions, Kang et al. reported that the strain state of QWs has also been affected by the electrical injection due to the electron accumulation in active regions^103^. A direct measurement technique was developed to study the stress variations of AlGaN MQWs under electrical injection. A tensile stress was found to be enhanced when the injecting current increases (Fig. 7c), thereby causing CH band to lift upward and the degree of polarization to decrease. It was revealed that the relaxation of tensile strain or the increase of compressive strain pulls the discrete quantum states of heavy holes and light holes back to the valence band maximum (VBM), thereby dramatically improving the total spontaneous emission rate (Fig. 7d). In 2021, Kang et al. proposed compressively strained (AlN)_8_/(GaN)_2_ nanorods by strain engineering digitally alloyed GaN well, thereby enabling the emission wavelength to reach 220 nm in the far-UVC with a higher transition probability from the heavy- and light hole bands. Moreover, they pushed the limits of QW structures based on AlGaN materials^104^.

This recent progress suggests that the control of the strain fields of high Al-content AlGaN MQWs is a promising way to improve the transverse electric (TE) polarized emission and increase the quantum efficiency in DUV optoelectronic devices.

## Manipulation of atomic orbital coupling

**Fig. 8:**
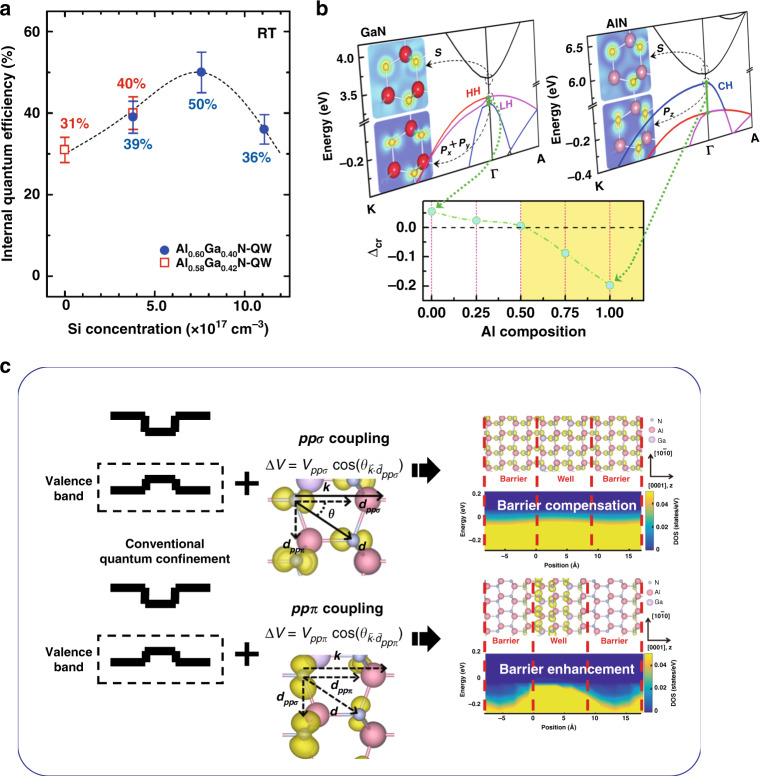
The orbital configuration and coupling in AlGaN. **a** The maximum value of the IQE at 300 K obtained from Si-doped AlGaN-QWs as a function of Si concentration in the well layers. **b** Band structure of GaN and AlN together with the crystal field splitting Δcr as a function of Al composition of Al_*x*_Ga_1*−x*_N. **c** Schematic contribution of the *ppσ* and *ppπ* coupling to the valence band offset in the conventional quantum model. Figures reproduced from **a** ref. ^105^, © 2011 AIP Publishing, **b** ref. ^108^, © 2013 John Wiley and Sons; **c** and **d**: ref. ^113^, © 2020 Springer Nature

In current modern optoelectronic devices, the IQE is closely related to the energy band structure in the active layer consisting of quantum structures, which directly derive from the quantum states. Strenuous efforts have been made to increase the IQE within the MQWs. In 2012, Murotani et al. reported that the minimization of the spatial separation between electron and hole wave functions can be achieved by reducing the thickness of the QW, thereby improving the radiation recombination probability^105^. Recent experimental works by Banal et al. and Bryan et al. have employed a moderate amount of Si doping into the QWs and barriers in various combinations to improve the quality of the well/barrier interfaces with a reduced density of point defects (Fig. 8a)^105–107^. Moreover, Grandusky et al. also pointed out that the Si-doped AlGaN QWs allow for the suppression of the band bending in QWs^23^. Thus, fundamental investigations on the underlying physics of the quantum states in MQWs under different structures and various fields become more crucial and important.

Aside from the challenges at the technical level, the orbital configuration of quantum levels in MQWs should be the most fundamental, which could directly influence the probability of particle transition between the band-edge states and thus the photon behaviors. In 2013, Lin et al. studied the optical anisotropy in AlGaN and recognized that the light emission polarized perpendicular to the *c*-axis is closely related to the near band-edge transitions occurring between the conduction bottom and the top of the valence bands^108^. The conduction band minimum (CBM) at Γ point is solely composed of *s-*orbitals with even symmetry along any axis going through its center. In previous study on the optical anisotropy in AlGaN, it was recognized that, in contrast to the Ga-rich AlGaN, the VBM is dominated by the CH band in Al-rich AlGaN instead of HH/LH bands (Fig. 8b)^108,109^. With respect to *c*-axis, the CH band at VBM is composed of *p*_*z*_-orbitals with odd symmetry. On account of parity selection rules, the interband transitions at the bandgap are readily accessible for TM polarized light (*E*⊥*c*) propagating laterally in the *c* plane^110^. With higher transition energy, the available TE polarized light emitting outward from the *c* plane is much less, and this limits the LEE. In 2017, investigations by Chen et al. demonstrated that the CH-band emission at the band edge exhibited abnormal radiative interband transitions in contrast to the HH/LH-band emission, which is sensitive to external electric fields^111^. The abnormal hole deconfinement has been understood by considering the orbital configuration in the dispersive CH-band. Basically, in 2010, Hirayama et al. pointed out that the conventional confinement was based on the continuous potential derived from the heterostructure band offset^112^, which did not consider the atomic orbital role in the quantum confinement. As the quantum structures go down to the atomic scale, the lattice discontinuity become increasingly unavoidable in a more micro perspective, where the pivotal role of the orbital intercoupling is at the forefront. The orientational sensitivity of the active valence *p*-states becomes strong along the confining direction in the quantum structures^113^.

The induced energy gain has magnitudes matched to the band offset with changes in sign depending on the microscopic details in the orbital inter-coupling. Therefore, the barrier potential for the confinement is determined by the joint effect of orbital inter-coupling and the band offset. Recent studies of the orbital-state coupling revealed that the head-on coupling between *p-*orbitals yielding the *ppσ* coupling is favorable for the band offset compensation, while the sideways coupling of parallel *p*-orbitals causing the *ppπ* coupling is favorable for the barrier enhancement, as shown in Fig. 8c. The energy gain with changes in sign contributing to the compensation or enhancement of the band offset crucially depends on the orbital coupling orientation with respect to the quantum confinement. By varying the confining directions, the orbital engineering has been proposed to customized quantum confinement to tailor luminance intensity. The interaction between the charge confinement of the hole band and the orbital coupling modulating is demonstrated by inclining the well plane via constructing the well on the semipolar and nonpolar planes implemented in the microrods. The higher emission intensity from the QW on the nonpolar plane is confirmed by localized cathodoluminescence. The concept of orbital engineering provides a fertile base for designing new materials through the combination of numerous orbital configurations, as well as size-dependent electrical and optical properties of quantum structures caused by quantum confinement effects.

## Manipulation of photonic fields

In optoelectronic devices, light generation, emission, absorption, and propagation are all highly correlated to the photonic field. For DUV devices, it has been widely concerned that the low LEE strongly hinders the rapid improvement of the light output power. It is well recognized that the emission light in Al-rich AlGaN QWs is primarily dominated by TM polarization, which propagates mainly towards the sidewall. The LED fabrication for sidewall collection is very difficult. In the past decade, researches have been widely conducted on these related issues, e.g., the enhancement of light extraction, the switching of the light propagation modes, the emission enhancement by electromagnetic coupling, etc. Therefore, it is demonstrated that the photonic field plays a crucial role in effectively operating the photon behavior and enhancing the photon extraction.

To increase the LEE, many efforts have been made to operate the light propagations, including the development of novel transparent electrode materials, the introduction of high reflective electrodes, and the fabrication of photonic nanostructures. As we know, the DUV light could be easily absorbed by most matters due to the high energy and short wavelength. Generally, materials that are transparent to DUV light have a wide bandgap and possess insulating properties. Such materials are rare, and the pursuit of novel materials and advanced techniques is difficult.

**Fig. 9:**
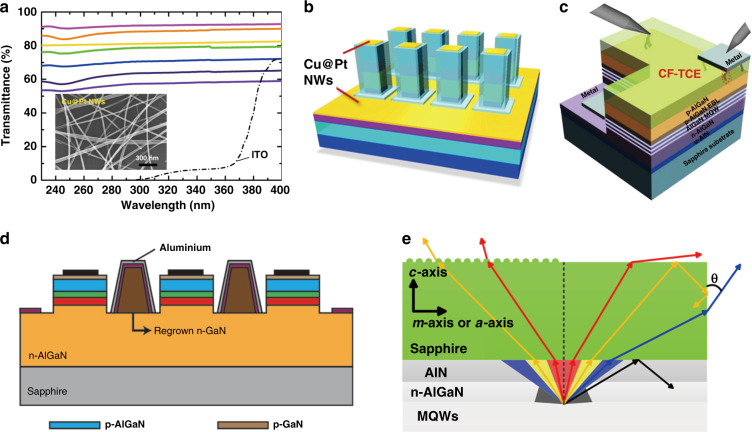
Transparent and reflective electrodes for UV bands. **a** UV−vis transmission spectra for Cu NWs electrodes and ITO. The inset is the SEM image of Cu@Pt NWs network. **b** Schematic illustration of the complete structure of AlGaN-based DUV LED with Cu@Pt NWs transparent electrodes. **c** Schematic of the AlGaN-based UV LED with the conductive-filament transparent electrodes. **d** Schematic of DUV LED with regrown n-GaN-based Al mirror between the narrow mesa stripe structures. **e** Light propagation characteristics in DUV LED with moth-eye microstructure fabricated on the backside of a sapphire substrate. Figures reproduced from **a** ref. ^114^, © 2013 Springer Nature; **b** ref. ^25^ © 2020, American Chemical Society; **c** ref. ^116^, © 2013 John Wiley and Sons; **d** ref. ^117^, © 2015 Springer Nature; **e** ref. ^42^, © 2018 American Chemical Society

In 2013, Cai et al. successfully synthesized via solution method the ultrafine and super long Cu nanowires (NWs) as transparent electrodes and revealed the unique full and high transparency (higher than 90%) from DUV to near-infrared region (200–3000 nm) (Fig. 9a)^114^. The light transmission mechanism on NWs network electrode has been regarded as a photon penetration and diffraction through the empty space between NWs, which is in the absence of matter. Because of the almost absorption-free feature to photons at any wavelength, the transparency of Cu NWs electrode for DUV light appears extremely high. In 2016, core–shell structured Cu NWs with various metal shells were achieved by one-pot method^115^, and together with broad work-function tunability, Cu@Pt NWs transparent electrodes led to the efficient ohmic contact to AlGaN-based DUV LEDs (275 nm) with enhanced light output power (wall plug efficiency of 3%) by 103% (Fig. 9b)^25^. Another novel technique, which was proposed by Kim et al. in 2014 and named electrical breakdown technique, which achieved DUV transparent conductive electrodes by forming conductive filaments (CFs) through SiN_*x*_, AlN thin film, AlN rod array, or embedded insulating ITO to the *p*-type AlGaN (Fig. 9c)^116^. Ohmic type contact with the high transparency (higher than 90%) to DUV light has been obtained.

Reflective electrodes have been introduced to the top surface and sidewall of DUV LED mesas to enhance the light extraction. In 2015, Kim et al. proposed the sidewall emission-enhanced DUV LEDs with three-dimensional Al reflectors between the narrow mesa stripes^117^, which could effectively enhance the light extraction of TM light (Fig. 9d). In 2017, Takano et al. proposed the combination of the AlGaN:Mg *p*-type contact layer and the Rh mirror electrode, which has significantly increased the output power and the EQE (more than 20%) of DUV-LEDs^28^. On the other hand, in 2018, Chen et al. introduced a moth-eye microstructure on the backside of a sapphire substrate and demonstrated an optical polarization of high degree (more than 80%) as well as the enhanced TE mode light intensity, thereby resulting in a doubled LEE (Fig. 9e)^42^.

**Fig. 10:**
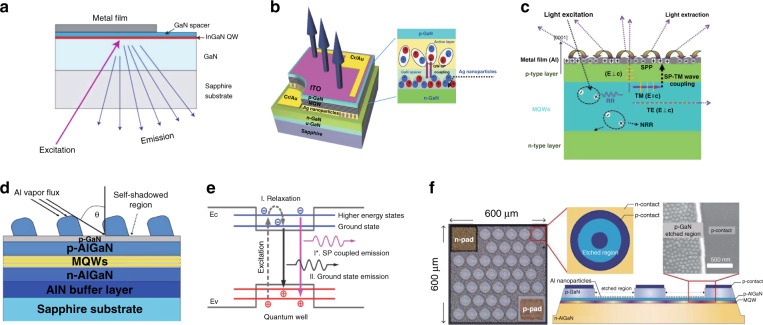
Versatile metallic structures embedded into the LEDs and the schematically SP-enhanced illustration. **a** The sample structure of the SP-enhanced InGaN quantum wells by deposition of different metallic films. **b** Schematic 3D representation of an InGaN/GaN LED with embedded Ag nanoparticles. Schematic illustration (**c**) of the SP enhanced and **d** the LSP enhanced DUV LEDs by ultrathin Al film and Al nanoparticles, respectively. **e** A schematic diagram showing the transition mechanisms for ground state emission and SP-coupled hot carrier emission in an AlGaN MQW structure and **f** the DUV LED through LSP coupling resonance with a high-density array of Al nanoparticles formed in the etched region. Figures reproduced from **a** ref. ^120^, © 2004 Springer Nature; **b** ref. ^119^, © 2008 John Wiley and Sons; **c** ref. ^125^, © 2012 Springer Nature; **d** ref. ^126^, © 2014 Springer Nature; **e** ref. ^127^, © 2014 John Wiley and Sons; **f** ref. ^130^, © 2020 ACS Publications

Recent developments of surface plasmon polaritons (SPPs) have opened the new way to improve the efficiency and performance of solid-state light sources because of their capability of controlling light propagation at subwavelength scale^118^. However, most efforts have been devoted to surface-plasmon- (SP-) enhanced light emission at visible wavelengths for LEDs since 1990 (Fig. 10a, b)^118–122^. In 2010, Lin et al. reported an efficient enhancement of UV-light emission from AlGaN/GaN SQW by depositing various metallic thin films onto the epitaxial layers^123^. In the case of AlGaN/GaN SQW excited from the top, the emission was enhanced via SP–QW coupling in the presence of both Ag and Al thin films. However, they only predicted that Al film could be extended to enhancement in DUV region.

Usually, in a DUV LED structure with high Al-content Al_*x*_Ga_1*−x*_N alloys as the active region, the emitted photons in the active layers can only partially escape from the top and bottom surfaces when inside an escape cone, with the emission polarized perpendicular to the *c*-axis (TE waves). The dominant emissions would be polarized parallel to the *c*-axis (TM waves), thus the rest part of TE waves outside the escape cone, and the entire TM waves would transmit along the direction perpendicular to the *c-*axis, thereby implying that DUV emission can no longer be extracted easily. In light of the fact that only the fundamental TM mode (*p*-polarized wave) is able to excite SPPs with the resulting matched momentum between the metal/semiconductor interfaces^124^, in 2012, Gao et al. demonstrated the extracted light emission of DUV LEDs can be enhanced by using the metallic Al thin film for SP coupling (Fig. 10c)^125^. For ultrathin Al layer deposited on the top of the DUV LEDs, parallel to the TM waves dominate in high Al-content Al_*x*_Ga_1*−x*_N alloys, while the top surface of the Al layer is not perfectly flat. Associated with the same frequency of TM waves and SPPs bridged by Al/AlGaN interface, the SPPs will propagate through the Al layer and thereupon recombine and emit light efficiently. Through these steps, the light extraction towards the top surface of DUV LEDs is enhanced by the SP-TM wave coupling. The light extraction was increased by 217% and 136% in peak photoluminescence intensity with a wavelength of 294 and 282 nm, respectively. Furthermore, the cathodoluminescence measurements provided evidence that the IQE of the DUV LEDs coated with Al layer was not enhanced by SP-QW coupling, thus the extraction of DUV light towards the top should be significantly enhanced.

Thereafter, versatile Al metallic structures embedded into the DUV range were proposed for efficient SP-based enhancement. Huang et al. optimized the metallic Al thin layer with polygonal geometry Al nanoparticles by localized surface plasmon (LSP) resonance (Fig. 10d), both the top- and bottom-emission EL at a wavelength of 279 nm were effectively enhanced^126^. In the same year in 2014, Yin et al. demonstrated a maximum enhancement of 3.2-fold of the DUV emission by the coupling of the LSP from Al nanoparticle arrays with the hot excitons in AlGaN-based MQWs structure (Fig. 10e)^127^. In 2018, Su et al. reported the enhancements in different polarizations of DUV QWs by fabricating Al nanogratings on an epitaxial structure for introducing SP coupling^128^. Recently, numerical study by employing Al/Al_2_O_3_ core–shell nanoparticle on the *p*-GaN contact layer was performed, through the careful regulation of the size and depth of nanoparticles, the considerable improvement for emission characteristics of SP-enhanced in DUV 240–280 nm range was exhibited^129^. In 2020, Lee et al. presented a remarkable increase in the efficiency of Al_0.43_Ga_0.57_N/Al_0.50_Ga_0.50_N MQWs at 285 nm, enabled by coupling LSP resonance with a high-density array of Al nanoparticles. The resultant IQE for the DUV LED was increased by 57.7% (Fig. 10f)^130^.

In addition, extensive studies have been dedicated to extracting the DUV light from the devices, such as surface texturing^131–133^, substrate patterning^84^, anti-reflective coatings^134^, highly reflective mirrors on top of the *p*-(Al)GaN^135^ and on the inclined sidewalls along the edge of the square-shape active mesa^136,137^. In an attempt to couple lateral emission to the outward emission of the top surface, the photonic crystal based on nanostructure is designed with air voids, nanopillar, nanorod, and NW structures^138–141^. From the band engineering perspective, recent results by Lin et al. addressed the compensation operation of asymmetry implemented by introducing some additional asymmetric periodicities into the matrix material to balance the intrinsic optical anisotropy in Al-rich AlGaN^108^. Specifically, the compensation of the Δ_cr_ was successfully achieved by the superimposition of variable asymmetrical ultrathin SLs into the anisotropic AlGaN host with high Al content. The optical isotropy supporting the transmission of TE and TM light with the same energy is accessible in ultrathin GaN/AlN SLs allowing for higher light emission and extraction.

## Manipulation of polarization fields

Because they are non-centro-symmetric and have high-degree ionicity, wurtzite III-nitrides exhibit strong spontaneous and piezoelectric polarization effects, which induces a strong built-in internal polarization field along [0001] direction^142^. The polarization field causes the band bending in (QWs, which results in a redshift of the emission and an overlap reduction of the electron and hole wave-functions, commonly known as “Quantum Confined Stark Effect (QCSE)”. Finally, the QCSE limits the radiative efficiency of III-nitride light emitters^143–145^. Great efforts have been made to reduce or eliminate the polarization field of the QWs in active region through various techniques. Furthermore, the polarization field within the III-nitride quantum structures has also been manipulated to achieve a high free-hole concentration in *p*-type AlGaN.

For the reduction or elimination of polarization field, doping in the active region, polarization-matched AlGaInN barriers, and varying QWs thickness have been proposed. The Si-doping QWs are most widely used to screen the polarization field for InGaN-based LEDs^42,146,147^. In 2006, Huang et al. reported the shielding of the polarization field in the AlGaN LEDs by the *n*-type doping^148^. In 2012, Murotani et al. revealed that the IQE of Al-rich AlGaN QWs increased from 19% to 40% by doping the well layers^105^. In 2014, Zhuo et al. investigated theoretically the Si-doping effect of band bending and carrier distribution for GaN/AlN QWs. The spatial separation of electrons and holes in the case of Si-doped in the wells was greatly impressed, it was thus favorable for the increase of the radiative efficiency of DUV-LEDs^149^. Reducing the QW width is another method for suppressing the effects of the polarization field in QW. Hirayama et al. in 2008 exhibited that the utilization of a thin QW in the active region was beneficial to increase the IQE of AlGaN DUV-LEDs^150^. As another approach, there has been an effort to substitute the conventional GaN barriers with quaternary AlGaInN barriers^151^. The use of quaternary alloys enables the interface polarization charge to be tuned over a range of values while keeping the bandgap constant. Therefore, the polarization-matched quaternary barriers can be realized with appropriately designed, which leads to less polarization electric field and improvement of the device performance.

In contrast to QWs in active region where the polarization field decreases the radiative efficiency, the polarization field is beneficial for *p*-type doping. Mg is the only known viable *p*-type dopant of III-nitride semiconductors^40^. However, it shows large activation energy (465–758 meV in AlN) in III-nitride semiconductors^40^, thereby only a small fraction of the dopant are ionized at room temperature. A large number of approaches, including SLs structure of *p*-type AlGaN and polarization-induced hole doping, have been proposed to assist the ionization of Mg acceptors by leveraging the polarization engineering^152,153^.

**Fig. 11:**
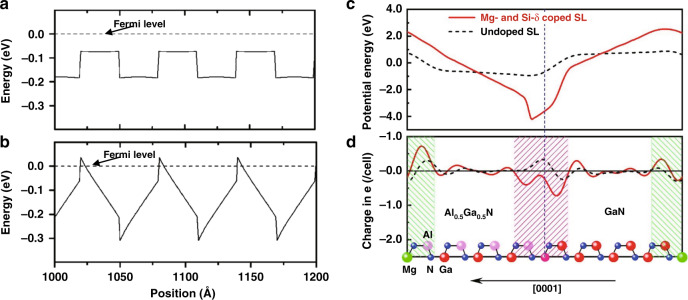
The polarization field effect on the energy band structures of Mg-doped AlGaN SLs. Calculated valence band diagram for Mg-doped Al_0.2_Ga_0.8_N/GaN SL in which the thickness of each layer is *L* = 30 Å, shown without (**a**) and with the polarization fields taken into account (**b**). The macroscopic averaged electrostatic potential (**c**) and macroscopic averaged differential charge density (**d**) of Mg- and Si-δ-codoped (solid lines) and undoped (dash lines) Al_0.5_Ga_0.5_N/GaN SLs plotted in the [0001] direction normal to the interface. Figures reproduced from **a** and **b** ref. ^153^, © 1999 AIP Publishing; **c** and **d** ref. ^155^, © 2009 AIP Publishing

Generally, the *p*-type AlGaN SLs consist of several thin *p*-AlGaN layers with alternating Al compositions, in which the periodic oscillation of the valence band edge induced by the polarization field can make the Mg-acceptor level close to the Fermi-level (Fig. 11a, b)^152,153^. The effective acceptor activation energy is thus reduced and high hole concentration can be achieved in SLs. In 1996, Schubert et al. first revealed in their theoretical work that the SLs doping can increase the acceptor activation efficiency by more than one order of magnitude^152^. In 1999, Kozodoy et al. demonstrated experimentally that the hole concentration was increased to 2.6 × 10^18^ cm^−3^ in uniformly doped Al_0.2_Ga_0.8_N/GaN SLs with optimal SL dimension, in which the effective acceptor activation energy was only 16 meV^153^. In 2001, Waldron et al. proposed a modulation-doped *p*-type AlGaN/GaN SLs to reduce the neutral impurity scattering in GaN well, the mobility was thus increased from that of 3 cm^2^ V^−1^ in the uniformly doped structure to 8.9 cm^2^ V^−1^^154^. To further increase Mg acceptor activation efficiency, in 2009, Li et al. proposed Mg- and Si-δ-codoped AlGaN SLs by introducing the monoatomic layer of Mg and Si at the different interfaces of SLs, respectively^155^. Because of the charge transferring from the Si-doped interface to Mg-doped interface, the internal electric fields in SLs were significantly intensified (Fig. 11c, d). Thus, the increased band bending caused the Mg acceptor level to be much closer to the Fermi-level. The Hall effect measurement results revealed that a hole concentration as high as 5.77 × 10^18^ cm^−3^ was achieved, which was twice that in modulation-doped SLs.

**Fig. 12:**
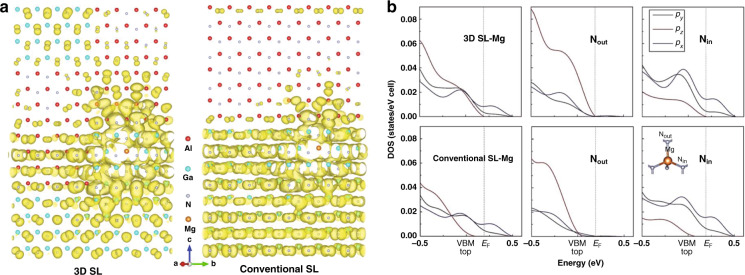
The charge distribution (orbitals) at the top of valance bands in SLs structures. **a** The isosurface of the valence states at the top of valance bands for Mg-doped 3D and conventional GaN/AlN SLs with *k* vector restricted to [0001]. **b** Decomposed DOS of *p*_*x*_, *p*_*y*_, and *p*_*z*_ of Mg and bonded N atoms in 3D SL and conventional SL. The N bonded with Mg lying in the *ab* plane and out of the plane is respectively denoted as N_in_ and N_out_ in the inset. Figures reproduced from ref. ^156^, © 2016 Springer Nature

Although 2D hole gases of high density can be formed in these SLs structures, they suffer from low conductivity along the *c*-axis. To enhance vertical hole conductivity, Zheng et al. proposed a novel three-dimensional (3D) Mg-doped SL in 2016^156^. The first-principle simulations indicated that the hole potential barrier along the *c*-axis significantly deceased in the 3D SL, thereby attributing to the stronger *p*_*z*_-hybridization between Mg and N. Therefore, the hole in the 3D SLs were more delocalized rather than concentrated in the well, compared to those in the conventional SLs (Fig. 12a). Further analysis of the site-decomposed density of states (DOS) of Mg and N atoms showed that the higher value in *p*_*z*_-DOS nearby the Fermi level of N atoms bonded with Mg was much higher (Fig. 12b), thereby producing higher concentration of vertical hole. Based on the theoretical results, *p*-type 3D Al_0.63_Ga_0.37_N/Al_0.51_Ga_0.49_N SLs were realized by adjusting the nitridation time at the initial growth stage of MOCVD. The hole concentration reached a value of 3.5 × 10^18^ cm^−3^, while the corresponding resistivity was as low as 0.7 Ω cm at room temperature, thereby exhibiting a conductivity improvement by 10 times in compared to that of conventional SLs.

**Fig. 13:**
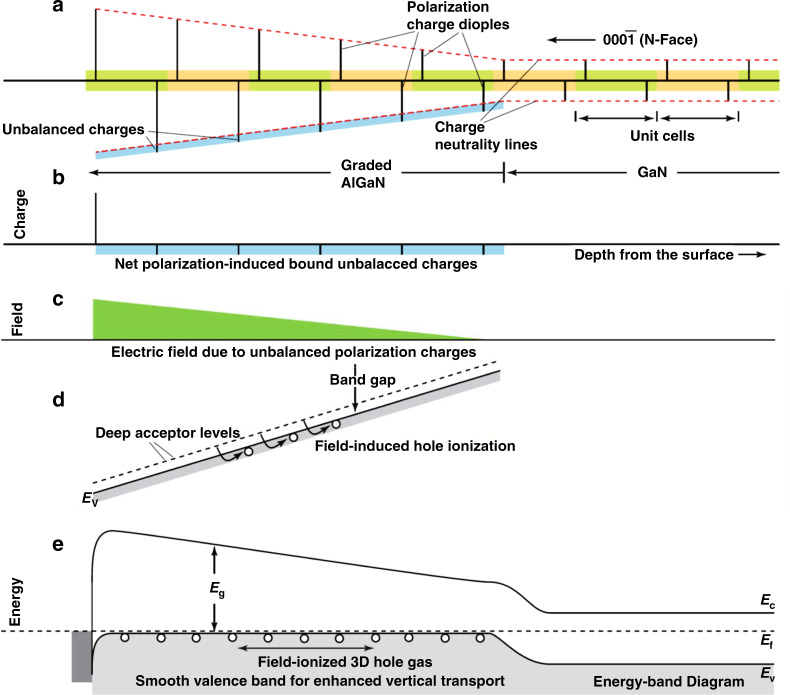
Schematic illustration of polarization-induced *p*-type doping in graded polar heterostructures. **a** Sheets of charge dipoles in every unit cell of the crystal. The net unbalanced polarization charge is shown in (**b**), which leads to the electric field in (**c**), and the energyband bending in the valence band in (**d**) if holes are not ionized. Field ionization of holes results in a steady-state energy-band diagram shown in (**e**), which highlights the smooth valence-band edge without any potential barriers for hole flow. *E*_f_, is the Fermi level; *E*_c_ and *E*_v_ are the conduction and valence-band edges, respectively; and *E*_g_ is the band gap. Figures reproduced from ref. ^157^, © 2010 AAAS

Polarization-induced hole doping is another approach for *p*-type doping in AlGaN. This method utilizes the gradually stacked polarization discontinuity in the Al-composition-graded AlGaN layer, which results in the formation of 3D bound charges. Thus, a built-in electric field is generated, which can activate the acceptor and make the valence band of AlGaN grading layer smoother to facilitate vertical hole transport, consequently, a 3D hole gas is generated (Fig. 13)^157^. This mechanism of polarization-induced hole formation was first proposed by Simon et al. in 2009^157^. A mobile 3D hole gas with a density of about 2 × 10^18^ cm^−3^ was achieved by linearly increasing Al content along the [000$\overline 1$] direction. Based on the concept of polarization-induced hole doping, many researchers have realized high-efficiency *p*-type doping in graded AlGaN. In 2010, Zhang et al. reported a hole concentration of ~2 × 10^18^ cm^−3^ in an AlGaN layer by decreasing the Al content from 0.3 to 0 along the [0001] direction^158^. In 2013, Li et al. realized polarization-induced hole doping on the order of ~10^18^ cm^−3^ in AlGaN layer with linearly graded Al content from 0.7 to 1 along with the [0001] direction by using Be as dopant^159^. In 2018, Yan et al. demonstrated that the hole concentration was increased by ~17 times in a linearly grading N-polar Al_*x*_Ga_1*−x*_N (*x* = 0–0.3) layer, compared to that of N-polar *p*-GaN^160^. Furthermore, Zhang et al. realized a 278.1 nm DUV laser diode for room temperature operation by employing a polarization-induced doping layer without intentional impurity^161^.

Apart from the free-carrier density, the efficient injection of holes into the active region is also intricately dependent on carrier mobility. Although a free-hole density of the order of 10^18^ cm^−3^ has been achieved in high Al-content AlGaN, the hole mobility is 10 times smaller than the electron mobility. In other words, a strong asymmetry between electron- and hole-transport still exists in DUV LEDs, thereby resulting in the electron overflow from the active region to the *p*-type layer without recombination as well as self-heating, which, in turn, leads to the efficiency droop^162,163^. Furthermore, the poor conductivity of *p*-type layers leads to higher contact and epilayer resistances and limits their current spreading length. This consequently causes the severe self-heating effect.

To block the electron overflow, *p*-AlGaN electron blocking layer (EBL) is typically employed, but it causes a penalty in operating voltage at the same time. Thus, a major effort is required to design the EBL structures. In 2014, Frank et al. proposed Mg-doped AlN/Al_0.7_Ga_0.3_N electron blocking heterostructures with optimized AlN thickness to ensure charge carrier injection and suppress the electron leakage in sub-250 nm DUV LEDs^164^ In 2015, Fan et al. adopted inverted-V-shaped graded Al composition EBL to reduce the efficiency droop of DUV LED. Liu et al. proposed an AlGaN SLs with varying barriers as the EBL of DUV LED, thereby causing the efficiency droop decreased from 80.8% to 28.8%^165^. In 2017 and 2018, Zhang et al. modified the barrier height for EBL by utilizing *p*-Al_0.60_Ga_0.40_N (L2)/Al_0.50_Ga_0.50_N/*p*-Al_0.60_Ga_0.40_N (L1) EBL and grading the alloy composition respectively, to guarantees a smooth hole injection into the active region^166,167^. In 2019, Lang et al. adopted an Al-composition and thickness-graded multiple quantum barriers structure as polarization-modulated EBL to enhance the carrier transport in DUV LED^168^. Furthermore, hole reservoir layers with different structures, such as graded AlGaN SLs^169^, Al-composition-graded layer^112^, and inverted-V-shaped quantum barrier^170^, showed significant suppression in the efficiency droop of DUV LEDs.

To eliminate the self-heating, some attempts have been conducted for DUV LEDs. In 2002, Shatalov et al. demonstrated that the current crowding in DUV LEDs could be alleviated by using the strip-geometry *p*-electron^171^. In 2004, Adivarhan et al. proposed a 10 × 10 array of interconnected micropixel structure to reduce both the device series resistance and the thermal impedance^172^. In 2009, they demonstrated that the vertical current conduction geometry of a device could also effectively reduce thermal impedance^173^. In 2018 Che et al. designed a *p*-AlGaN/*n*-AlGaN/*p*-AlGaN structured current spreading layer in the *p*-type hole injection layer^174^. In 2019 Chun et al. improved the current spreading of DUV LEDs by modulating the resistivity in the *n*-AlGaN layer^175^. Zhang et al. proposed a honeycomb hole-shaped structure of an electrode to improve the current spreading for 280-nm DUV LED^176^.

This progress demonstrates that the proper design of MQWs, *p*-type structure, EBL, hole reservoir layers, current spreading layer, and device geometry play an important role in increasing the efficiency of DUV LED.

## Conclusion and outlook

In summary, we have reviewed recent progress in the development of novel concepts and techniques on AlGaN-based LEDs and summarized that multiple physical fields could build the toolkit for effectively controlling and tailoring the crucial properties of nitride quantum structures. By manipulating the fields of chemical potentials, the short-period GaN/AlN SLs that are atomically flat and abrupt interfaces can be realized for the replacement of high-Al-content AlGaN alloys. To release misfit strain during heteroepitaxial growth and heterostructural construction, different approaches such as the ELOG, the multi-period SLs inserting layers, and the van-der-Waals epitaxial growth have been adopted. Furthermore, the strain fields within the AlGaN QWs can be intentionally managed to improve the TE polarized emission and increase the quantum efficiency in DUV LEDs. To improve the IQE of AlGaN MQWs, the optimization of orbital-state coupling was proved significant in enhancing the combination of numerous orbital configurations as well as size-dependent electrical and optical properties. Meanwhile, the polarization field could be reduced by methods such as doping in the active region, polarization-matched AlGaInN barriers, and varying QWs thickness for improving the radiative efficiency. In contrast, the polarization field could also be manipulated to achieve a high free-hole concentration in *p*-type AlGaN. The photonic field plays a crucial role in effectively operating the photon behavior and enhancing the photon extraction. Various techniques, including novel transparent electrodes, high reflective electrodes, photonic nanostructures, surface plasmon coupling, and surface texturing, have been developed to operate the light propagations. Moreover, the TE-polarized dominated emission could be enhanced by band engineering and thus lead to increased LEE.

**Fig. 14:**
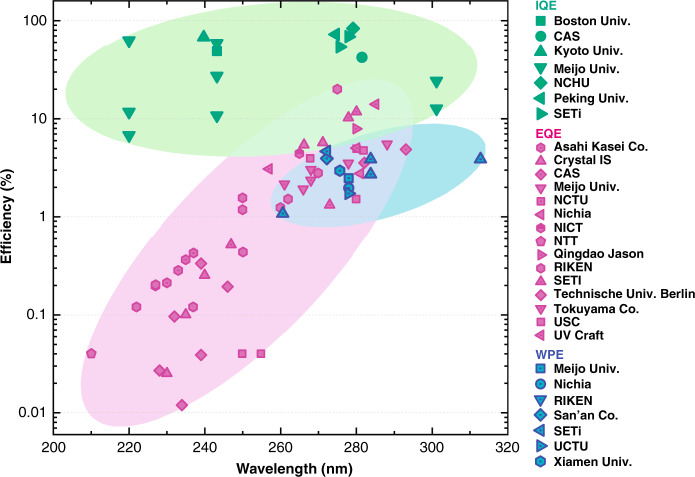
EQEs (pink dots), WPEs (blue dots) and IQEs (green dots) for commercial and laboratorial DUV LEDs. Data points obtained from refs. ^24,25,28,29,31,32,52,56,106,112,131,164,177–192^.

There are a couple of challenges ahead, from the bottom substrate up to the top electrodes, for approaching high-efficiency, high-power, and high-reliability DUV LEDs. In contrast to the conventional epilayer, novel quantum structures in a scaling-down size and in more complicated configuration exhibit their unique advantages by their implantation into different parts of the device structure. Brand new solutions have been found to overcome existing challenges. Furthermore, the fundamental physical fields acting on these quantum structures have gradually built up a clear system stepwise, which seems to provide multiple keys for opening or turning the corresponding problems. Past developments have achieved great enhancements to the performance of AlGaN-based DUV LEDs. Fig. 14 summarizes the EQEs, wall-plug efficiencies (WPEs), and IQEs of the available data of commercial and laboratory DUV LEDs in a wavelength range between 200 and 310 nm^24,25,28,29,31,32,52,56,106,112,131,164,177–192^. The EQE and WPE obviously decrease rapidly as the wavelength gets shorter. However, despite the large deviation, the IQE seems much higher than EQE, this indicates a big room for improvement of devices performance. It is believed that the WPE of commercially or laboratorially available DUV LEDs in the 265–280 nm emission bands will increase by over 20% in the very near future, e.g., by 2025.

## Supplementary information

Reproduction permissions for Figure 3

Reproduction permissions for Figure 4

Reproduction permissions for Figure 5

Reproduction permissions for Figure 6

Reproduction permissions for Figure 7

Reproduction permissions for Figure 8

Reproduction permissions for Figure 9

Reproduction permissions for Figure 10

Reproduction permissions for Figure 11

Reproduction permissions for Figure 12

Reproduction permissions for Figure 13
