# Supplementary material for: Multiple fields manipulation on nitride material structures in ultraviolet light-emitting diodes
Source: Light Sci Appl. 2021 Jun 16;10:129. doi: 10.1038/s41377-021-00563-0 (PMC8206881; doi:10.1038/s41377-021-00563-0)
Supplement: Supplementary file 1 — Reproduction permissions for Figure 3 [file 41377_2021_563_MOESM1_ESM.pdf]

Defect Suppression in AlN Epilayer Using Hierarchical Growth Units

Author: Qinqin Zhuang, Wei Lin, Weihuang Yang, et al

Publication: The Journal of Physical Chemistry C

Publisher: American Chemical Society

Date: Jul 1, 2013

Copyright © 2013, American Chemical Society

PERMISSION/LICENSE IS GRANTED FOR YOUR ORDER AT NO CHARGE

This type of permission/license, instead of the standard Terms & Conditions, is sent to you because no fee is being charged for your order. Please note the following:

- Permission is granted for your request in both print and electronic formats, and translations.
- If figures and/or tables were requested, they may be adapted or used in part.
- Please print this page for your records and send a copy of it to your publisher/graduate school.
- Appropriate credit for the requested material should be given as follows: "Reprinted (adapted) with permission from (COMPLETE REFERENCE CITATION). Copyright (YEAR) American Chemical Society." Insert appropriate information in place of the capitalized words.
- One-time permission is granted only for the use specified in your request. No additional uses are granted (such as derivative works or other editions). For any other uses, please submit a new request.

If credit is given to another source for the material you requested, permission must be obtained from that source.

BACK | CLOSE WINDOW

© 2020 Copyright - All Rights Reserved | Copyright Clearance Center, Inc. | Privacy statement | Terms and Conditions
Comments? We would like to hear from you. E-mail us at customercare@copyright.com
