# Supplementary material for: Multiple fields manipulation on nitride material structures in ultraviolet light-emitting diodes
Source: Light Sci Appl. 2021 Jun 16;10:129. doi: 10.1038/s41377-021-00563-0 (PMC8206881; doi:10.1038/s41377-021-00563-0)
Supplement: Supplementary file 3 — Reproduction permissions for Figure 5 [file 41377_2021_563_MOESM3_ESM.pdf]

AIP PUBLISHING LICENSE
TERMS AND CONDITIONS

May 09, 2021

This Agreement between jinchai li ("You") and AIP Publishing ("AIP Publishing") consists of your license details and the terms and conditions provided by AIP Publishing and Copyright Clearance Center.

License Number	5065091143506
License date	May 09, 2021
Licensed Content Publisher	AIP Publishing
Licensed Content Publication	Applied Physics Letters
Licensed Content Title	High-spatial-resolution strain measurements by Auger electron spectroscopy in epitaxial-lateral-overgrowth GaN
Licensed Content Author	Duanjun Cai, Fuchun Xu, Junyong Kang, et al
Licensed Content Date	May 23, 2005
Licensed Content Volume	86
Licensed Content Issue	21

Type of Use	Journal/Magazine
Requestor type	Author (original article)
Format	Print and electronic
Portion	Figure/Table
Number of figures/tables	2
Title of new article	Multiple Fields Manipulation on Nitride Material Structures in Ultraviolet Light-Emitting Diodes
Lead author	Jinchai Li
Title of targeted journal	Light: Science & Applications
Publisher	Springer Nature
Expected publication date	May 2021
Order reference number	81
Portions	Figure 1 and Figure 4
Requestor Location	jinchai li<br>422-19, Siming South road, Xiamen<br>Department of Physics, Xiamen University<br><br>Fujian Province, other 361005<br>China<br>Attn: jinchai li

Total 0.00 USD

Terms and Conditions

AIP Publishing -- Terms and Conditions: Permissions Uses

AIP Publishing hereby grants to you the non-exclusive right and license to use and/or distribute the Material according to the use specified in your order, on a one-time basis, for the specified term, with a maximum distribution equal to the number that you have ordered. Any links or other content accompanying the Material are not the subject of this license.

1. You agree to include the following copyright and permission notice with the reproduction of the Material:"Reprinted from [FULL CITATION], with the permission of AIP Publishing." For an article, the credit line and permission notice must be printed on the first page of the article or book chapter. For photographs, covers, or tables, the notice may appear with the Material, in a footnote, or in the reference list.
2. If you have licensed reuse of a figure, photograph, cover, or table, it is your responsibility to ensure that the material is original to AIP Publishing and does not contain the copyright of another entity, and that the copyright notice of the figure, photograph, cover, or table does not indicate that it was reprinted by AIP Publishing, with permission, from another source. Under no circumstances does AIP Publishing purport or intend to grant permission to reuse material to which it does not hold appropriate rights.
   You may not alter or modify the Material in any manner. You may translate the Material into another language only if you have licensed translation rights. You may not use the Material for promotional purposes.
3. The foregoing license shall not take effect unless and until AIP Publishing or its agent, Copyright Clearance Center, receives the Payment in accordance with Copyright Clearance Center Billing and Payment Terms and Conditions, which are incorporated herein by reference.
4. AIP Publishing or Copyright Clearance Center may, within two business days of granting this license, revoke the license for any reason whatsoever, with a full refund payable to you. Should you violate the terms of this license at any time, AIP Publishing, or Copyright Clearance Center may revoke the license with no refund to you. Notice of such revocation will be made using the contact information provided by you. Failure to receive such notice will not nullify the revocation.
5. AIP Publishing makes no representations or warranties with respect to the Material. You agree to indemnify and hold harmless AIP Publishing, and their officers, directors, employees or agents from and against any and all claims arising out of your use of the Material other than as specifically authorized herein.

6. The permission granted herein is personal to you and is not transferable or assignable without the prior written permission of AIP Publishing. This license may not be amended except in a writing signed by the party to be charged.
7. If purchase orders, acknowledgments or check endorsements are issued on any forms containing terms and conditions which are inconsistent with these provisions, such inconsistent terms and conditions shall be of no force and effect. This document, including the CCC Billing and Payment Terms and Conditions, shall be the entire agreement between the parties relating to the subject matter hereof.

This Agreement shall be governed by and construed in accordance with the laws of the State of New York. Both parties hereby submit to the jurisdiction of the courts of New York County for purposes of resolving any disputes that may arise hereunder.

V1.2

Questions? customercare@copyright.com or +1-855-239-3415 (toll free in the US) or +1-978-646-2777.

AIP PUBLISHING LICENSE
TERMS AND CONDITIONS

May 08, 2021

This Agreement between jinchai li ("You") and AIP Publishing ("AIP Publishing") consists of your license details and the terms and conditions provided by AIP Publishing and Copyright Clearance Center.

License Number	5064170076935
License date	May 08, 2021
Licensed Content Publisher	AIP Publishing
Licensed Content Publication	Applied Physics Letters
Licensed Content Title	Dislocation density reduction via lateral epitaxy in selectively grown GaN structures
Licensed Content Author	Tsvetanka S. Zheleva, Ok-Hyun Nam, Michael D. Bremser, et al
Licensed Content Date	Oct 27, 1997
Licensed Content Volume	71
Licensed Content Issue	17
Type of Use	Journal/Magazine
Requestor type	Author/Researcher/Scientist

Format	Electronic
Portion	Figure/Table
Number of figures/tables	1
Title of new article	Multiple Fields Manipulation on Nitride Material Structures in Ultraviolet Light-Emitting Diodes
Lead author	Jinchai Li
Title of targeted journal	Light: Science & Applications
Publisher	Springer Nature
Expected publication date	May 2021
Order reference number	78
Portions	Figure5
Requestor Location	jinchai li<br>422-19, Siming South road, Xiamen<br>Department of Physics, Xiamen University<br><br>Fujian Province, other 361005<br>China<br>Attn: jinchai li
Total	0.00 USD

Terms and Conditions

AIP Publishing -- Terms and Conditions: Permissions Uses

AIP Publishing hereby grants to you the non-exclusive right and license to use and/or distribute the Material according to the use specified in your order, on a one-time basis, for the specified term, with a maximum distribution equal to the number that you have ordered. Any links or other content accompanying the Material are not the subject of this license.

1. You agree to include the following copyright and permission notice with the reproduction of the Material:"Reprinted from [FULL CITATION], with the permission of AIP Publishing." For an article, the credit line and permission notice must be printed on the first page of the article or book chapter. For photographs, covers, or tables, the notice may appear with the Material, in a footnote, or in the reference list.
2. If you have licensed reuse of a figure, photograph, cover, or table, it is your responsibility to ensure that the material is original to AIP Publishing and does not contain the copyright of another entity, and that the copyright notice of the figure, photograph, cover, or table does not indicate that it was reprinted by AIP Publishing, with permission, from another source. Under no circumstances does AIP Publishing purport or intend to grant permission to reuse material to which it does not hold appropriate rights.
   You may not alter or modify the Material in any manner. You may translate the Material into another language only if you have licensed translation rights. You may not use the Material for promotional purposes.
3. The foregoing license shall not take effect unless and until AIP Publishing or its agent, Copyright Clearance Center, receives the Payment in accordance with Copyright Clearance Center Billing and Payment Terms and Conditions, which are incorporated herein by reference.
4. AIP Publishing or Copyright Clearance Center may, within two business days of granting this license, revoke the license for any reason whatsoever, with a full refund payable to you. Should you violate the terms of this license at any time, AIP Publishing, or Copyright Clearance Center may revoke the license with no refund to you. Notice of such revocation will be made using the contact information provided by you. Failure to receive such notice will not nullify the revocation.
5. AIP Publishing makes no representations or warranties with respect to the Material. You agree to indemnify and hold harmless AIP Publishing, and their officers, directors, employees or agents from and against any and all claims arising out of your use of the Material other than as specifically authorized herein.
6. The permission granted herein is personal to you and is not transferable or assignable without the prior written permission of AIP Publishing. This license may not be amended except in a writing signed by the party to be charged.
7. If purchase orders, acknowledgments or check endorsements are issued on any forms containing terms and conditions which are inconsistent with these provisions, such inconsistent terms and conditions shall be of no force and effect. This document, including the CCC Billing and

Payment Terms and Conditions, shall be the entire agreement between the parties relating to the subject matter hereof.

This Agreement shall be governed by and construed in accordance with the laws of the State of New York. Both parties hereby submit to the jurisdiction of the courts of New York County for purposes of resolving any disputes that may arise hereunder.

V1.2

Questions? customercare@copyright.com or +1-855-239-3415 (toll free in the US) or +1-978-646-2777.

AIP PUBLISHING LICENSE
TERMS AND CONDITIONS

May 08, 2021

This Agreement between jinchai li ("You") and AIP Publishing ("AIP Publishing") consists of your license details and the terms and conditions provided by AIP Publishing and Copyright Clearance Center.

License Number	5064171460511
License date	May 08, 2021
Licensed Content Publisher	AIP Publishing
Licensed Content Publication	Applied Physics Letters
Licensed Content Title	282-nm AlGaN-based deep ultraviolet light-emitting diodes with improved performance on nano-patterned sapphire substrates
Licensed Content Author	Peng Dong, Jianchang Yan, Junxi Wang, et al
Licensed Content Date	Jun 17, 2013
Licensed Content Volume	102
Licensed	24

Content Issue	
Type of Use	Journal/Magazine
Requestor type	Author (original article)
Format	Electronic
Portion	Figure/Table
Number of figures/tables	2
Title of new article	Multiple Fields Manipulation on Nitride Material Structures in Ultraviolet Light-Emitting Diodes
Lead author	Jinchai Li
Title of targeted journal	Light: Science & Applications
Publisher	Springer Nature
Expected publication date	May 2021
Order reference number	84
Portions	Figure2(a) and Figure 3 on page 2
Requestor Location	jinchai li<br>422-19, Siming South road, Xiamen<br>Department of Physics, Xiamen University<br><br>Fujian Province, other 361005

China
Attn: jinchai li

Total 0.00 USD

Terms and Conditions

AIP Publishing -- Terms and Conditions: Permissions Uses

AIP Publishing hereby grants to you the non-exclusive right and license to use and/or distribute the Material according to the use specified in your order, on a one-time basis, for the specified term, with a maximum distribution equal to the number that you have ordered. Any links or other content accompanying the Material are not the subject of this license.

1. You agree to include the following copyright and permission notice with the reproduction of the Material:"Reprinted from [FULL CITATION], with the permission of AIP Publishing." For an article, the credit line and permission notice must be printed on the first page of the article or book chapter. For photographs, covers, or tables, the notice may appear with the Material, in a footnote, or in the reference list.
2. If you have licensed reuse of a figure, photograph, cover, or table, it is your responsibility to ensure that the material is original to AIP Publishing and does not contain the copyright of another entity, and that the copyright notice of the figure, photograph, cover, or table does not indicate that it was reprinted by AIP Publishing, with permission, from another source. Under no circumstances does AIP Publishing purport or intend to grant permission to reuse material to which it does not hold appropriate rights.
   You may not alter or modify the Material in any manner. You may translate the Material into another language only if you have licensed translation rights. You may not use the Material for promotional purposes.
3. The foregoing license shall not take effect unless and until AIP Publishing or its agent, Copyright Clearance Center, receives the Payment in accordance with Copyright Clearance Center Billing and Payment Terms and Conditions, which are incorporated herein by reference.
4. AIP Publishing or Copyright Clearance Center may, within two business days of granting this license, revoke the license for any reason whatsoever, with a full refund payable to you. Should you violate the terms of this license at any time, AIP Publishing, or Copyright Clearance Center may revoke the license with no refund to you. Notice of such revocation will be made using the contact information provided by you. Failure to receive such notice will not nullify the revocation.
5. AIP Publishing makes no representations or warranties with respect to the Material. You agree to indemnify and hold harmless AIP Publishing,

and their officers, directors, employees or agents from and against any and all claims arising out of your use of the Material other than as specifically authorized herein.

6. The permission granted herein is personal to you and is not transferable or assignable without the prior written permission of AIP Publishing. This license may not be amended except in a writing signed by the party to be charged.
7. If purchase orders, acknowledgments or check endorsements are issued on any forms containing terms and conditions which are inconsistent with these provisions, such inconsistent terms and conditions shall be of no force and effect. This document, including the CCC Billing and Payment Terms and Conditions, shall be the entire agreement between the parties relating to the subject matter hereof.

This Agreement shall be governed by and construed in accordance with the laws of the State of New York. Both parties hereby submit to the jurisdiction of the courts of New York County for purposes of resolving any disputes that may arise hereunder.

V1.2

Questions? customercare@copyright.com or +1-855-239-3415 (toll free in the US) or +1-978-646-2777.

AIP PUBLISHING LICENSE
TERMS AND CONDITIONS

May 08, 2021

This Agreement between jinchai li ("You") and AIP Publishing ("AIP Publishing") consists of your license details and the terms and conditions provided by AIP Publishing and Copyright Clearance Center.

License Number	5064180496552
License date	May 08, 2021
Licensed Content Publisher	AIP Publishing
Licensed Content Publication	Applied Physics Letters
Licensed Content Title	High quality 10.6 μm AlN grown on pyramidal patterned sapphire substrate by MOCVD
Licensed Content Author	Hanling Long, Jiangnan Dai, Yi Zhang, et al
Licensed Content Date	Jan 28, 2019
Licensed Content Volume	114
Licensed Content Issue	4
Type of Use	Journal/Magazine
Requestor type	Original Author Representative

Format	Print and electronic
Portion	Figure/Table
Number of figures/tables	1
Title of new article	Multiple Fields Manipulation on Nitride Material Structures in Ultraviolet Light-Emitting Diodes
Lead author	Jinchai Li
Title of targeted journal	Light: Science & Applications
Publisher	Springer Nature
Expected publication date	May 2021
Order reference number	85
Portions	Figure 3
Requestor Location	jinchai li<br>422-19, Siming South road, Xiamen<br>Department of Physics, Xiamen University<br><br>Fujian Province, other 361005<br>China<br>Attn: jinchai li
Total	0.00 USD

Terms and Conditions

AIP Publishing -- Terms and Conditions: Permissions Uses

AIP Publishing hereby grants to you the non-exclusive right and license to use and/or distribute the Material according to the use specified in your order, on a one-time basis, for the specified term, with a maximum distribution equal to the number that you have ordered. Any links or other content accompanying the Material are not the subject of this license.

1. You agree to include the following copyright and permission notice with the reproduction of the Material:"Reprinted from [FULL CITATION], with the permission of AIP Publishing." For an article, the credit line and permission notice must be printed on the first page of the article or book chapter. For photographs, covers, or tables, the notice may appear with the Material, in a footnote, or in the reference list.
2. If you have licensed reuse of a figure, photograph, cover, or table, it is your responsibility to ensure that the material is original to AIP Publishing and does not contain the copyright of another entity, and that the copyright notice of the figure, photograph, cover, or table does not indicate that it was reprinted by AIP Publishing, with permission, from another source. Under no circumstances does AIP Publishing purport or intend to grant permission to reuse material to which it does not hold appropriate rights.
   You may not alter or modify the Material in any manner. You may translate the Material into another language only if you have licensed translation rights. You may not use the Material for promotional purposes.
3. The foregoing license shall not take effect unless and until AIP Publishing or its agent, Copyright Clearance Center, receives the Payment in accordance with Copyright Clearance Center Billing and Payment Terms and Conditions, which are incorporated herein by reference.
4. AIP Publishing or Copyright Clearance Center may, within two business days of granting this license, revoke the license for any reason whatsoever, with a full refund payable to you. Should you violate the terms of this license at any time, AIP Publishing, or Copyright Clearance Center may revoke the license with no refund to you. Notice of such revocation will be made using the contact information provided by you. Failure to receive such notice will not nullify the revocation.
5. AIP Publishing makes no representations or warranties with respect to the Material. You agree to indemnify and hold harmless AIP Publishing, and their officers, directors, employees or agents from and against any and all claims arising out of your use of the Material other than as specifically authorized herein.
6. The permission granted herein is personal to you and is not transferable or assignable without the prior written permission of AIP Publishing. This license may not be amended except in a writing signed by the party to be charged.
7. If purchase orders, acknowledgments or check endorsements are issued on any forms containing terms and conditions which are inconsistent with these provisions, such inconsistent terms and conditions shall be of no force and effect. This document, including the CCC Billing and

Payment Terms and Conditions, shall be the entire agreement between the parties relating to the subject matter hereof.

This Agreement shall be governed by and construed in accordance with the laws of the State of New York. Both parties hereby submit to the jurisdiction of the courts of New York County for purposes of resolving any disputes that may arise hereunder.

V1.2

Questions? customercare@copyright.com or +1-855-239-3415 (toll free in the US) or +1-978-646-2777.
