# Supplementary material for: Multiple fields manipulation on nitride material structures in ultraviolet light-emitting diodes
Source: Light Sci Appl. 2021 Jun 16;10:129. doi: 10.1038/s41377-021-00563-0 (PMC8206881; doi:10.1038/s41377-021-00563-0)
Supplement: Supplementary file 5 — Reproduction permissions for Figure 7 [file 41377_2021_563_MOESM5_ESM.pdf]

AIP PUBLISHING LICENSE
TERMS AND CONDITIONS

May 08, 2021

---

This Agreement between jinchai li ("You") and AIP Publishing ("AIP Publishing") consists of your license details and the terms and conditions provided by AIP Publishing and Copyright Clearance Center.

| | |
|---|---|
| License Number | 5064210302397 |
| License date | May 08, 2021 |
| Licensed Content Publisher | AIP Publishing |
| Licensed Content Publication | Applied Physics Letters |
| Licensed Content Title | Effect of strain and barrier composition on the polarization of light emission from AlGaN/AlN quantum wells |
| Licensed Content Author | J. E. Northrup, C. L. Chua, Z. Yang, et al |
| Licensed Content Date | Jan 9, 2012 |
| Licensed Content Volume | 100 |
| Licensed Content Issue | 2 |
| Type of Use | Journal/Magazine |

| | |
|---|---|
| Requestor type | Author (original article) |
| Format | Print and electronic |
| Portion | Figure/Table |
| Number of figures/tables | 1 |
| Title of new article | Multiple Fields Manipulation on Nitride Material Structures in Ultraviolet Light-Emitting Diodes |
| Lead author | Jinchai Li |
| Title of targeted journal | Light: Science & Applications |
| Publisher | Springer Nature |
| Expected publication date | May 2021 |
| Order reference number | 97 |
| Portions | Figure 3 on page 2 |
| Requestor Location | jinchai li<br>422-19, Siming South road, Xiamen<br>Department of Physics, Xiamen University<br><br>Fujian Province, other 361005<br>China<br>Attn: jinchai li |
| Total | 0.00 USD |

Terms and Conditions

AIP Publishing -- Terms and Conditions: Permissions Uses

AIP Publishing hereby grants to you the non-exclusive right and license to use and/or distribute the Material according to the use specified in your order, on a one-time basis, for the specified term, with a maximum distribution equal to the number that you have ordered. Any links or other content accompanying the Material are not the subject of this license.

1. You agree to include the following copyright and permission notice with the reproduction of the Material:"Reprinted from [FULL CITATION], with the permission of AIP Publishing." For an article, the credit line and permission notice must be printed on the first page of the article or book chapter. For photographs, covers, or tables, the notice may appear with the Material, in a footnote, or in the reference list.
2. If you have licensed reuse of a figure, photograph, cover, or table, it is your responsibility to ensure that the material is original to AIP Publishing and does not contain the copyright of another entity, and that the copyright notice of the figure, photograph, cover, or table does not indicate that it was reprinted by AIP Publishing, with permission, from another source. Under no circumstances does AIP Publishing purport or intend to grant permission to reuse material to which it does not hold appropriate rights.
   You may not alter or modify the Material in any manner. You may translate the Material into another language only if you have licensed translation rights. You may not use the Material for promotional purposes.
3. The foregoing license shall not take effect unless and until AIP Publishing or its agent, Copyright Clearance Center, receives the Payment in accordance with Copyright Clearance Center Billing and Payment Terms and Conditions, which are incorporated herein by reference.
4. AIP Publishing or Copyright Clearance Center may, within two business days of granting this license, revoke the license for any reason whatsoever, with a full refund payable to you. Should you violate the terms of this license at any time, AIP Publishing, or Copyright Clearance Center may revoke the license with no refund to you. Notice of such revocation will be made using the contact information provided by you. Failure to receive such notice will not nullify the revocation.
5. AIP Publishing makes no representations or warranties with respect to the Material. You agree to indemnify and hold harmless AIP Publishing, and their officers, directors, employees or agents from and against any and all claims arising out of your use of the Material other than as specifically authorized herein.
6. The permission granted herein is personal to you and is not transferable or assignable without the prior written permission of AIP Publishing. This license may not be amended except in a writing signed by the party to be charged.

7. If purchase orders, acknowledgments or check endorsements are issued on any forms containing terms and conditions which are inconsistent with these provisions, such inconsistent terms and conditions shall be of no force and effect. This document, including the CCC Billing and Payment Terms and Conditions, shall be the entire agreement between the parties relating to the subject matter hereof.

This Agreement shall be governed by and construed in accordance with the laws of the State of New York. Both parties hereby submit to the jurisdiction of the courts of New York County for purposes of resolving any disputes that may arise hereunder.

V1.2

**Questions? customercare@copyright.com or +1-855-239-3415 (toll free in the US) or +1-978-646-2777.**

AIP PUBLISHING LICENSE
TERMS AND CONDITIONS

May 08, 2021

---

This Agreement between jinchai li ("You") and AIP Publishing ("AIP Publishing") consists of your license details and the terms and conditions provided by AIP Publishing and Copyright Clearance Center.

| | |
|---|---|
| License Number | 5064210564490 |
| License date | May 08, 2021 |
| Licensed Content Publisher | AIP Publishing |
| Licensed Content Publication | Applied Physics Letters |
| Licensed Content Title | Strain dependence on polarization properties of AlGaN and AlGaN-based ultraviolet lasers grown on AlN substrates |
| Licensed Content Author | Zachary Bryan, Isaac Bryan, Seiji Mita, et al |
| Licensed Content Date | Jun 8, 2015 |
| Licensed Content Volume | 106 |
| Licensed Content Issue | 23 |

| | |
|---|---|
| Type of Use | Journal/Magazine |
| Requestor type | Author (original article) |
| Format | Print and electronic |
| Portion | Figure/Table |
| Number of figures/tables | 1 |
| Title of new article | Multiple Fields Manipulation on Nitride Material Structures in Ultraviolet Light-Emitting Diodes |
| Lead author | Jinchai Li |
| Title of targeted journal | Light: Science & Applications |
| Publisher | Springer Nature |
| Expected publication date | May 2021 |
| Order reference number | 98 |
| Portions | Figure 3(a) on page4 |
| Requestor Location | jinchai li<br>422-19, Siming South road, Xiamen<br>Department of Physics, Xiamen University<br><br>Fujian Province, other 361005<br>China<br>Attn: jinchai li |

Total 0.00 USD

Terms and Conditions

AIP Publishing -- Terms and Conditions: Permissions Uses

AIP Publishing hereby grants to you the non-exclusive right and license to use and/or distribute the Material according to the use specified in your order, on a one-time basis, for the specified term, with a maximum distribution equal to the number that you have ordered. Any links or other content accompanying the Material are not the subject of this license.

1. You agree to include the following copyright and permission notice with the reproduction of the Material:"Reprinted from [FULL CITATION], with the permission of AIP Publishing." For an article, the credit line and permission notice must be printed on the first page of the article or book chapter. For photographs, covers, or tables, the notice may appear with the Material, in a footnote, or in the reference list.
2. If you have licensed reuse of a figure, photograph, cover, or table, it is your responsibility to ensure that the material is original to AIP Publishing and does not contain the copyright of another entity, and that the copyright notice of the figure, photograph, cover, or table does not indicate that it was reprinted by AIP Publishing, with permission, from another source. Under no circumstances does AIP Publishing purport or intend to grant permission to reuse material to which it does not hold appropriate rights.
   You may not alter or modify the Material in any manner. You may translate the Material into another language only if you have licensed translation rights. You may not use the Material for promotional purposes.
3. The foregoing license shall not take effect unless and until AIP Publishing or its agent, Copyright Clearance Center, receives the Payment in accordance with Copyright Clearance Center Billing and Payment Terms and Conditions, which are incorporated herein by reference.
4. AIP Publishing or Copyright Clearance Center may, within two business days of granting this license, revoke the license for any reason whatsoever, with a full refund payable to you. Should you violate the terms of this license at any time, AIP Publishing, or Copyright Clearance Center may revoke the license with no refund to you. Notice of such revocation will be made using the contact information provided by you. Failure to receive such notice will not nullify the revocation.
5. AIP Publishing makes no representations or warranties with respect to the Material. You agree to indemnify and hold harmless AIP Publishing, and their officers, directors, employees or agents from and against any and all claims arising out of your use of the Material other than as specifically authorized herein.

6. The permission granted herein is personal to you and is not transferable or assignable without the prior written permission of AIP Publishing. This license may not be amended except in a writing signed by the party to be charged.
7. If purchase orders, acknowledgments or check endorsements are issued on any forms containing terms and conditions which are inconsistent with these provisions, such inconsistent terms and conditions shall be of no force and effect. This document, including the CCC Billing and Payment Terms and Conditions, shall be the entire agreement between the parties relating to the subject matter hereof.

This Agreement shall be governed by and construed in accordance with the laws of the State of New York. Both parties hereby submit to the jurisdiction of the courts of New York County for purposes of resolving any disputes that may arise hereunder.

V1.2

**Questions? customercare@copyright.com or +1-855-239-3415 (toll free in the US) or +1-978-646-2777.**

×

# Effect of electrical injection-induced stress on interband transitions in high Al content AlGaN MQWs

J. Zheng, J. Li, Z. Zhong, W. Lin, L. Chen, K. Li, X. Wang, C. Chou, S. Li and J. Kang, *RSC Adv.*, 2017, **7**, 55157
**DOI:** 10.1039/C7RA10440E

This article is licensed under a Creative Commons Attribution-NonCommercial 3.0 Unported Licence. Material from this article can be used in other publications provided that the correct acknowledgement is given with the reproduced material and it is not used for commercial purposes.

Reproduced material should be attributed as follows:

- For reproduction of material from NJC:
  [Original citation] - Published by The Royal Society of Chemistry (RSC) on behalf of the Centre National de la Recherche Scientifique (CNRS) and the RSC.
- For reproduction of material from PCCP:
  [Original citation] - Published by the PCCP Owner Societies.
- For reproduction of material from PPS:
  [Original citation] - Published by The Royal Society of Chemistry (RSC) on behalf of the European Society for Photobiology, the European Photochemistry Association, and RSC.
- For reproduction of material from all other RSC journals:
  [Original citation] - Published by The Royal Society of Chemistry.

Information about reproducing material from RSC articles with different licences is available on our Permission Requests page.

×
