# Supplementary material for: Multiple fields manipulation on nitride material structures in ultraviolet light-emitting diodes
Source: Light Sci Appl. 2021 Jun 16;10:129. doi: 10.1038/s41377-021-00563-0 (PMC8206881; doi:10.1038/s41377-021-00563-0)
Supplement: Supplementary file 10 — Reproduction permissions for Figure 12 [file 41377_2021_563_MOESM10_ESM.pdf]

Improved p-type conductivity in Al-rich AlGaN using multidimensional Mg-doped superlattices

SPRINGER NATURE

Author: T. C. Zheng et al

Publication: Scientific Reports

Publisher: Springer Nature

Date: Feb 24, 2016

Copyright © 2016, The Author(s)

Creative Commons

This is an open access article distributed under the terms of the Creative Commons CC BY license, which permits unrestricted use, distribution, and reproduction in any medium, provided the original work is properly cited.

You are not required to obtain permission to reuse this article.

To request permission for a type of use not listed, please contact Springer Nature

© 2021 Copyright - All Rights Reserved | Copyright Clearance Center, Inc. | Privacy statement | Terms and Conditions

Comments? We would like to hear from you. E-mail us at customercare@copyright.com
